# Smoking Genes: A Case–Control Study of Dopamine Transporter Gene (*SLC6A3*) and Dopamine Receptor Genes (*DRD1*, *DRD2* and *DRD3*) Polymorphisms and Smoking Behaviour in a Malay Male Cohort

**DOI:** 10.3390/biom10121633

**Published:** 2020-12-03

**Authors:** Abu Bakar Ruzilawati, Md Asiful Islam, Siti Khariem Sophia Muhamed, Imran Ahmad

**Affiliations:** 1 Department of Pharmacology, School of Medical Sciences, Universiti Sains Malaysia, Kubang Kerian 16150, Kelantan, Malaysia; ruzila@usm.my (A.B.R.); karensofia812@gmail.com (S.K.S.M.); 2 Department of Haematology, School of Medical Sciences, Universiti Sains Malaysia, Kubang Kerian 16150, Kelantan, Malaysia; asiful@usm.my; 3 Department of Family Medicine, School of Medical Sciences, Universiti Sains Malaysia, Kubang Kerian 16150, Kelantan, Malaysia

**Keywords:** smoking behaviour, dopamine transporter, dopamine receptor, gene, polymorphism

## Abstract

Dopamine receptor and dopamine transporter genes polymorphisms have been associated with cigarette smoking behaviour in different populations. The aim of this case–control study was to evaluate polymorphisms in the dopamine transporter gene (*SLC6A3* (rs27072)) and the dopamine receptor genes (*DRD1* (rs686), *DRD2* (rs1800497) and *DRD3* (rs7653787)) and their contribution to smoking behaviour in a Malay male population. We identified 476 participants over the age of 18 years comprising 238 smokers and 238 non-smokers. Information such as age, height, weight, body mass index, systolic and diastolic blood pressures, marital status, and smoking status of close family members were taken. For the genetic study, we genotyped four genes (*SLC6A3* (rs27072), *DRD1* (rs686), *DRD2* (rs1800497) and *DRD3* (rs7653787)) using the polymerase chain reaction–restriction fragment length polymorphism method and further confirmed our findings with sequencing. Dopamine receptor genes (*DRD1*, *DRD2* and *DRD3*) were found to be associated with smoking behaviour in a Malay male population. The dopamine transporter gene (*SLC6A3*) did not show this association. Significant differences were observed between smokers’ and non-smokers’ age, systolic blood pressure, marital status and family members who smoke. Smoking behaviour is significantly influenced by genetic variations of *DRD1*, *DRD2* and *DRD3* in a Malay male population.

## 1. Introduction

Tobacco smoking is highly addictive because of nicotine. The role of genetics in nicotine addiction supports the known associations of smoking behaviour and cessation with genetic variations [1,2,3,4]. Nicotine continuously exerts “rewarding effects” by increasing the production of dopamine in the nucleus accumbens of the brain and thus alters the mesolimbic dopamine pathway [3,5,6]. Interestingly, nicotine addiction has been significantly associated with polymorphisms in the dopamine receptor or dopamine transporter genes, which play roles in the dopamine pathway [7,8]. These genes encode for the proteins involved in the synthesis or metabolism of dopamine [8].

For instance, the dopamine transporter gene *SLC6A3* (previously known as *DAT1*; genomic location: 5p15.33, 52,651 bases) encodes a sodium- and chloride-dependent neurotransmitter that pumps the neurotransmitter dopamine out of the synaptic cleft back into the cytosol where vesicular monoamine transporter 2 confiscates it into vesicles for storage and further release [9]. The 3′ untranslated region (UTR) of *SLC6A3* contains 3–11 copies of a 40 bp tandem repeat. Variation in the number of repeats is associated with dependence on alcohol and cocaine and protection against nicotine dependence [10,11]. Polymorphisms in the coding regions of *SLC6A3* (rs27072) have been associated with bipolar disorder and tobacco addiction [12,13,14]. Some dopamine receptor genes, such as *DRD1* [15], *DRD2* [16,17], *DRD3* [18,19], *DRD4* [18] and *DRD5* [20], have been associated with smoking behaviour in various populations. All five of the dopamine receptor genes are G-protein-coupled receptors.

*DRD1* (genomic location: 5q35.2 4147 bases) encodes for the D1 subtype of the five (D1–D5) dopamine receptors, the most abundant dopamine receptor in the central nervous system (CNS) [21]. It functions by stimulating adenylyl cyclase and activating cyclic AMP-dependent protein kinases. The D1 subtype also mediates behavioural disorders, such as pathological gambling, schizophrenia, Tourette’s syndrome, and amphetamine addiction [22,23,24,25]. Polymorphisms in this gene (rs686) have been associated with depressive symptoms, alcohol dependence, bipolar disorder, and nicotine dependence [26,27,28].

*DRD2*, previously known as *Taq1A* (genomic location: 11q23.2, 66,097 bases), encodes for the D2 subtype of the dopamine receptor and functions by inhibiting adenylyl cyclase activity. *DRD2* has been associated with some psychological disorders, such as pathological gambling, schizophrenia, and cocaine dependence, [29] and is the major receptor for most antipsychotic drugs. Specific polymorphisms in this gene (rs1800497) have been associated with mood disorders, migraine, and nicotine dependence [30,31].

*DRD3* (genomic location: 3q13.31, 71,611 bases) encodes the D3 subtype of the dopamine receptor that inhibits adenylyl cyclase. Genetic variations have been associated with different neurological abnormalities, such as tremors, schizophrenia, Parkinson’s disease, and drug addiction [32,33,34]. Polymorphisms in this gene (rs7653787) have been associated with nicotine dependence [35].

In Malaysia, the prevalence of cigarette smoking is approximately 22.5% [36]. Among the major three ethnic groups, smoking was found to be highly prevalent among Malays [37]. This study aimed to evaluate the association of gene polymorphisms in the dopamine transporter *SLC6A3* (rs27072) and dopamine receptors *DRD1* (rs686), *DRD2* (rs1800497) and *DRD3* (rs7653787) with smoking behaviour in a Malay male population.

## 2. Materials and Methods

### 2.1. Participants

Overall, 476 participants participated in this case–control study, including smokers (*n* = 238) and non-smokers (*n* = 238) as defined by their self-reported smoking status. The participants were Malay males between 18 and 50 years of age. The participants were classified as smokers based on whether they currently smoke cigarettes and had smoked >100 cigarettes in their lifetime as per the National Health Interview Survey (NHIS) [38]. Non-smokers identified themselves if they were not active smokers and had smoked <100 cigarettes in the past. Nicotine dependence was assessed using a validated Malay version of the six-item Fagerstrom Test for Nicotine Dependence [39]. Basic information such as age, height, weight, body mass index, systolic and diastolic blood pressures, marital status, and smoking status of close family members were taken. Ethics approval was obtained from the Universiti Sains Malaysia (USM/JEPeM/14110480).

### 2.2. Genetic Study

We collected venous blood (3 mL) from the participants for the genetic study. Genomic DNA was isolated from whole blood using a DNA extraction kit (G-spin™ Total DNA Extraction, iNtRON Biotechnology, Seongnam, South Korea). The genotyping of the four genes (*SLC6A3* (rs27072), *DRD1* (rs686), *DRD2* (rs1800497) and *DRD3* (rs7653787)) was performed using the polymerase chain reaction (PCR)–restriction fragment length polymorphism method in a thermal cycler (Eppendorf MasterCycler Nexus Gradient, Eppendorf, Germany). The PCR reaction was conducted in a final volume of 25 µL, followed by the digestion of the PCR products with certain restriction enzymes, including MspI, TaqIA, Cac8I and PsiI. The digested fragments were then analysed on 1% agarose gel using ethidium bromide staining followed by visualisation under ultraviolet light. Some PCR products were randomly selected for sequencing using the Applied Biosystems 3730 XL Genetic Analyser (Applied Biosystems, Foster City, CA, USA) and the BigDye^®^ Terminator v3.1 cycle sequencing kit (Invitrogen, Thermo Fisher Scientific, Waltham, MA, USA) was used for the sequence confirmation.

### 2.3. Data Analyses

Data were analysed using SPSS (version 24, IBM, Armonk, NY, USA) and Stata (version 16, StataCorp, College Station, TX, USA) software. The allele and genotype distributions were calculated to equilibrium using the Hardy–Weinberg equation (p^2^ + 2pq + q^2^ = 1). Non-parametric chi-square test was used to calculate the significance of the genotype and allele frequencies. Risk factors were assessed between cases and controls using both simple logistic regression (crude odds ratio (OR)) and multiple logistic regression analyses (adjusted OR). We considered a *p*-value of <0.05 to be statistically significant.

## 3. Results

### 3.1. Allele and Genotype Frequencies

We did not find a significant association either at the genotypic level (χ^2^ = 0.88, *p* = 0.64) or the allelic level (χ^2^ = 0.09, *p* = 0.75) of *SLC6A3* (rs27072) by comparing smokers to non-smokers (Table 1 and Table 2; Figures S1, S2 and S9). Among the smokers, a significant difference was found in the *DRD1* (rs686) genotype (χ^2^ = 54.70, *p* < 0.001) and allele frequencies (χ^2^ = 48.498, *p* < 0.001) compared with the non-smokers (Table 1; Figures S3, S4 and S10). The prevalence of AG genotype in *DRD1* (rs686) was significantly higher in smokers in compared with non-smokers (OR: 7.07, 95% CI: 3.71–13.42; *p* < 0.001; Table 2). We found a significant association of *DRD2* (rs1800497) with smoking at the genotypic level (*p* < 0.001); however, no significant association was observed at the allelic level (*p* = 0.36; Table 2; Figures S5, S6 and S11). At the genotypic level, A1/A2 was significantly associated with smoking behaviour (OR: 4.25, 95% CI: 2.24–8.05; *p* < 0.001; Table 2). In the case of *DRD3* (rs7653787), a significant difference was found at the genotypic level (χ^2^ = 12.81, *p* = 0.002) but not at the allelic level (χ^2^ = 2.62, *p* = 0.10) among the smokers (Table 1; Figures S7, S8 and S12). Interestingly, among the different *DRD3* genotypes (CC, CT and TT), the homozygous mutant CC was detected as a significantly protective genotype (OR: 0.11, 95% CI: 0.02–0.53, *p* = 0.006) in the smokers (Table 2).

### 3.2. Factors Associated with Smoking Behaviour

Interestingly, we found smoking behaviour to be associated with age (*p* < 0.001) and systolic blood pressure (*p* < 0.001; Table 2). Married males were significantly less likely to smoke compared with single males (*p* < 0.008); smoking behaviour was also significantly associated with family members who smoke (*p* < 0.001; Table 2).

## 4. Discussion

To the best of our knowledge, this is the first study to investigate the associations between the genetic polymorphisms within the dopamine transporter *SLC6A3* (rs27072) and dopamine receptors *DRD1* (rs686), *DRD2* (rs1800497) and *DRD3* (rs7653787) and smoking behaviour in the Malaysian population. Due to the social stigma of women smoking in Malaysia, we focused on males in our study population.

We found no significant association between the rs27072 polymorphism of *SLC6A3* and smoking behaviour in male Malay smokers. Several studies have explored the association between 3′ UTR polymorphism of *SLC6A3* and smoking behaviour [12,40,41,42,43]. However, only a few studies have investigated the smoking behaviour association with the rs27072 polymorphism. Ling et al. [44] and Breitling et al. [45] found that *SLC6A3* (rs27072) plays a significant role in the onset of smoking but is not significantly associated with nicotine dependence in Chinese and German populations, respectively. Recently, in a Russian population, no significant association was determined between *SLC6A3* (rs27072) and smoking behaviour [46]. However, a study on a Chinese population found that a higher *SLC6A3* score was associated with the possibility of quitting smoking among those with a lower level of nicotine dependence [47]. Therefore, the rs27072 polymorphism of *SLC6A3* may play a role in smoking behaviour. However, more studies with large sample sizes are warranted.

According to our study, the AG genotype of *DRD1* (rs686) was significantly associated with smoking behaviour (OR: 7.07, 95% CI: 3.71–13.42; *p* < 0.001) in the Malay male population. Novak et al. [35] reported that rs686 of *DRD1* was associated with the quantity of tobacco smoked (*p* = 0.005) in a Canadian cohort; thus, supporting the contribution of the rs686 polymorphism of *DRD1* in smoking behaviour. An interesting study in an American population [48] found that rs686 polymorphism in *DRD1* increased the risk of lung cancer in those who were exposed to second-hand smoke during childhood. Huang et al. [27] demonstrated that the rs686 polymorphism of *DRD1* is significantly associated with nicotine dependence in an African American population, which eventually affects the expression of *DRD1*. Therefore, our results are in line with previous reports, and the rs686 polymorphism of *DRD1* is significantly associated with smoking behaviour in our Malay male population.

With *DRD2* (rs1800497), the A1/A2 genotype was significantly associated with smoking behaviour in our cohort. In a few American cohorts, this gene was significantly associated with smoking behaviour [49,50,51]. Wilcox et al. [52] observed that the A1^+^ genotype (A1/A1, A1/A2) had more nicotine dependence compared with the A1^−^ (A2/A2) genotype. In contrast, a study with Japanese male smokers showed that the A2/A2 genotype had a higher risk of smoking behaviour [53]. A study on Czech patients with coronary heart disease found that the *DRD2* polymorphism (rs1800497) was significantly associated with failure to cease smoking [54]. Interestingly, *DRD2* polymorphism (rs1800497) did not have any relationship with smoking behaviour in Taiwanese men [55]. Although in a British cohort (*n* = 878), *DRD2* rs1800497 genotype was not associated with smoking cessation [56], in a one-year follow-up study on a German cohort (*n* = 577), *DRD2* rs1800497 genotype was found to be a predictor of smoking cessation [57]. Contradicting these findings, a second study on German subjects found no association between *DRD2* and heavy smoking [58]. These results suggest that in some populations, there is a significant association pattern between *DRD2* (rs1800497) and smoking behaviour.

Our study found that the TT genotype of *DRD3* (rs7653787) was under-represented in Malay smokers. A single prospective study on female participants established no relationship between *DRD3* polymorphism and smoking cessation when the participants were on d,1-fenfluramine [59]. In another study involving a cohort of 341 patients with schizophrenia [35], *DRD3* polymorphism (rs1025398) showed an association with the quantity of tobacco smoked (*p* = 0.002). Therefore, to the best of our knowledge, ours is the first study to demonstrate a protective role of rs7653787 polymorphism of *DRD3* in Malay smokers.

In our study, marital status was significantly associated with smoking behaviour; married participants were less likely to smoke than single participants, which is similar to the findings of Morton et al. [60]. However, some studies on Egyptian [61,62], Thai [63] and American [64] populations did not find any significant difference. Our study found that age was significantly different between smokers and non-smokers; smokers were younger, which is similar to a study on Thai males [60]. However, no significant difference was found in studies on Greek [65], Thai [60], Egyptian [62] and American [64] populations. Moreover, although our study determined that systolic blood pressure values were significantly lower in smokers, no such difference was observed in a Greek population [65]. We also observed a significant contribution to smoking behaviour when any of the family members smoked, which is in line with previous studies [66,67,68,69].

## 5. Conclusions

In summary, we found that gene polymorphisms in the dopamine receptors *DRD1*, *DRD2* and *DRD3* alter the dopaminergic response to nicotine, which contributes to smoking behaviour. A similar result was not observed with the dopamine transporter *SLC6A3*. We observed significant differences in age, systolic blood pressure, marital status and family members who smoke between Malay male smokers and non-smokers.

## Figures and Tables

**Table 1:** Genotype and allele frequencies of *SLC6A3* (rs27072), *DRD1* (rs686), *DRD2* (rs1800497) and *DRD3* (rs7653787) polymorphisms in smokers and non-smokers.

Genes (SNPs)	Smoking Status	Genotype (%)			*p*-Value ^a^	Allele		*p*-Value ^a^
*SLC6A3* (rs27072)		CC	CT	TT		C	T	
	Smoker	5 (2.1)	192 (80.7)	41 (17.2)	0.64	202 (42.4)	274 (57.6)	0.75
	Non-smoker	5 (2.1)	184 (77.3)	45 (20.6)		194 (41.5)	274 (58.5)	
*DRD1* (rs686)		AA	AG	GG		A	G	**<0.001**
	Smoker	157 (66.0)	81 (34.0)	0 (0.0)	**<0.001**	395 (83.0)	81 (34.0)	
	Non-smoker	222 (93.3)	16 (6.7)	0 (0.0)		460 (96.6)	16 (6.7)	
*DRD2* (rs1800497)		A1/A1	A1/A2	A2/A2		A1	A2	0.36
	Smoker	46 (19.3)	168 (70.6)	24 (10.1)	**<0.001**	260 (54.6)	216 (45.4)	
	Non-smoker	67 (28.2)	112 (47.1)	59 (24.8)		246 (51.7)	230 (48.3)	
*DRD3* (rs7653787)		CC	CT	TT		C	T	0.10
	Smoker	22 (9.2)	211 (88.7)	5 (2.1)	**0.002**	255 (53.6)	221 (46.4)	
	Non-smoker	7 (2.9)	216 (90.8)	15 (6.3)		230 (48.3)	246 (51.7)	

^a^ chi-square test. Significant *p*-values (*p* < 0.05) are in bold.

**Table 2:** Factors associated with smoking status.

Independent Variables	Crude OR ^a^ (95% CI)	Wald Statistics ^a^ (df)	*p*-Value ^a^	Adjusted OR ^b^ (95% CI)	Wald Statistics ^b^ (df)	*p*-Value ^b^
Age (year)	1.07 (1.05–1.09)	49.87 (1)	**<0.001**	1.09 (1.07–1.12)	56.60 (1)	<0.001
Height	0.42 (0.03–6.42)	0.39 (1)	0.531			
Weight	1.01 (1.00–1.02)	0.91 (1)	0.339			
Body mass index	1.03 (0.99–1.08)	1.85 (1)	0.174			
Systolic	0.98 (0.97–1.00)	7.17 (1)	0.007	0.96 (0.94–0.98)	21.49 (1)	<0.001
Diastolic	1.00 (0.98–1.02)	0.07 (1)	0.788			
Marital status						
Single	1.00 (Reference)					
Married	0.60 (0.41–0.88)	7.02 (1)	**0.008**			
Family members who smoke						
No	1.00 (Reference)					
Yes	0.11 (0.06–0.19)	59.33 (1)	**<0.001**			
*SLC6A3* (rs27072)						
CC	1.00 (Reference)					
TT	0.83 (0.23–3.10)	0.07 (1)	0.789			
CT	1.04 (0.30–3.66)	0.00 (1)	0.947			
*DRD1* (rs686)						
AA	1.00 (Reference)					
AG	7.16 (4.03–12.71)	45.20 (1)	**<0.001**	7.07 (3.71–13.42)	35.80 (1)	**<0.001**
*DRD2* (rs1800497)						
A1/A1	1.00 (Reference)					
A2/A2	1.69 (0.92–3.09)	2.88 (1)	0.090	1.94 (0.94–4.01)	19.60 (1)	**<0.001**
A1/A2	3.69 (2.17–6.27)	23.17 (1)	**<0.001**	4.25 (2.24–8.05)		
*DRD3* (rs7653787)						
TT	1.00 (Reference)					
CC	9.43 (2.51–35.37)	11.07 (1)	**0.001**	0.11 (0.02–0.53)	7.71 (1)	**0.006**
CT	2.93(1.05–8.21)	4.19 (1)	**0.041**	0.38 (0.14–1.02)		

OR: odds ratio. Significant *p*-values (*p* < 0.05) are in bold. ^a^ Simple logistic regression. ^b^ Multiple logistic regression.

## Supplementary Materials

The following are available online at https://www.mdpi.com/2218-273X/10/12/1633/s1, Figure S1: PCR product (460 bp) of *SLC6A3* gene (rs27072), Figure S2: RFLP result of *SLC6A3* gene (rs27072), Figure S3: PCR product (460 bp) of *DRD1* gene (rs686), Figure S4: RFLP result of *DRD1* gene (rs686), Figure S5: PCR product (300 bp) of *DRD2* gene (rs1800497), Figure S6: RFLP result of *DRD2* gene (rs1800497), Figure S7: PCR product (488 bp) of *DRD3* (rs7653787), Figure S8: RFLP result of *DRD3* gene (rs7653787), Figure S9: Sequencing analysis of *SLC6A3* gene (rs27072) (A) the chromatograms of *SLC6A3* gene (rs27072) homozygous wild type sequence and (B) *SLC6A3* gene (rs27072) heterozygous mutant sequence, Figure S10: Sequencing analysis of *DRD1* (rs686) (A) the chromatograms of *DRD1* gene (rs686) homozygous wild type sequence and (B) *DRD1* gene (rs686) heterozygous mutant sequence, Figure S11: Sequencing analysis of *DRD2* (rs1800497) (A) the chromatograms of *DRD2* gene (rs1800497) homozygous wild type sequence and (B) *DRD2* gene (rs1800497) heterozygous mutant sequence, Figure S12: Sequencing analysis of *DRD3* (rs7653787) (A) the chromatograms of *DRD3* gene (rs7653787) homozygous wild type sequence and (B) *DRD3* gene (rs7653787) heterozygous mutant sequence.

## References

[1] Tibuakuu M., Kamimura D., Kianoush S., DeFilippis A.P., Al Rifai M., Reynolds L.M., White W.B., Butler K.R., Mosley T.H., Turner S.T. (2017). The association between cigarette smoking and inflammation: The Genetic Epidemiology Network of Arteriopathy (GENOA) study. PLoS ONE.

[2] Sheu M.-J., Hsieh M.-J., Chou Y.-E., Wang P.-H., Yeh C.-B., Yang S.-F., Lee H.-L., Liu Y.-F. (2017). Effects of ADAMTS14 genetic polymorphism and cigarette smoking on the clinicopathologic development of hepatocellular carcinoma. PLoS ONE.

[3] Li S., Wang Q., Pan L., Yang X., Li H., Jiang F., Zhang N., Han M., Jia C. (2017). The association of environmental, individual factors, and dopamine pathway gene variation with smoking cessation. Psychol. Health Med..

[4] Benowitz N.L. (2010). Nicotine addiction. N. Engl. J. Med..

[5] David S.P., Munafò M.R. (2008). Genetic variation in the dopamine pathway and smoking cessation. Pharmacogenomics.

[6] Ericson M., Löf E., Stomberg R., Söderpalm B. (2009). The Smoking Cessation Medication Varenicline Attenuates Alcohol and Nicotine Interactions in the Rat Mesolimbic Dopamine System. J. Pharmacol. Exp. Ther..

[7] Dani J.A. (2003). Roles of dopamine signaling in nicotine addiction. Mol. Psychiatry.

[8] Herman A.I., DeVito E.E., Jensen K.P., Sofuoglu M. (2014). Pharmacogenetics of nicotine addiction: Role of dopamine. Pharmacogenomics.

[9] Baeuchl C., Chen H.-Y., Su Y.-S., Hämmerer D., Klados M.A., Li S.-C. (2019). Interactive effects of dopamine transporter genotype and aging on resting-state functional networks. PLoS ONE.

[10] Hiemstra M., Engels R.C.M.E., Barker E.D., Van Schayck O.C.P., Otten R. (2013). Smoking-Specific Parenting and Smoking Onset in Adolescence: The Role of Genes from the Dopaminergic System (*DRD2*, *DRD4*, *DAT1* Genotypes). PLoS ONE.

[11] Choi H.D., Shin W.G., Duck C.H., Gyoon S.W. (2016). Meta-analysis update of association between dopamine transporter *SLC6A3* gene polymorphism and smoking cessation. J. Health Psychol..

[12] Siemińska A., Buczkowski K., Jassem E., Niedoszytko M., Tkacz E. (2009). Influences of polymorphic variants of *DRD2* and *SLC6A3* genes, and their combinations on smoking in Polish population. BMC Med. Genet..

[13] Ma Y., Yuan W., Cui W., Li M.D. (2015). Meta-analysis reveals significant association of 3′-UTR VNTR in *SLC6A3* with smoking cessation in Caucasian populations. Pharm. J..

[14] Pinsonneault J.K., Han D.D., Burdick K.E., Kataki M., Bertolino A., Malhotra A.K., Gu H.H., Sadee W. (2011). Dopamine Transporter Gene Variant Affecting Expression in Human Brain is Associated with Bipolar Disorder. Neuropsychopharmacology.

[15] Lee W., Ray R., Bergen A.W., Swan G.E., Thomas P.D., Tyndale R.F., Benowitz N.L., Lerman C., Conti D.V. (2012). *DRD1* associations with smoking abstinence across slow and normal nicotine metabolizers. Pharm. Genom..

[16] Munafò M.R., Timpson N.J., David S.P., Ebrahim S., Lawlor D.A. (2009). Association of the *DRD2* gene *Taq1A* polymorphism and smoking behavior: A meta-analysis and new data. Nicotine Tob. Res..

[17] Vieira G., Cavalli J., Gonçalves E.C.D., Braga S.F.P., Ferreira R.S., Santos A.R., Cola M., Raposo N.R.B., Capasso R., Dutra R.C. (2020). Antidepressant-Like Effect of Terpineol in an Inflammatory Model of Depression: Involvement of the Cannabinoid System and D2 Dopamine Receptor. Biomolecules.

[18] Vandenbergh D.J., O’Connor R.J., Grant M.D., Jefferson A.L., Vogler G.P., Strasser A.A., Kozlowski L.T. (2007). Dopamine receptor genes (*DRD2*, *DRD3* and *DRD4*) and gene? Gene interactions associated with smoking-related behaviors. Addict. Biol..

[19] Bono F., Mutti V., Fiorentini C., Missale C. (2020). Dopamine D3 Receptor Heteromerization: Implications for Neuroplasticity and Neuroprotection. Biomolecules.

[20] Sullivan P.F., Neale M.C., Silverman M.A., Harris-Kerr C., Myakishev M.V., Wormley B., Webb B.T., Ma Y., Kendler K.S., Straub R.E. (2001). An association study of *DRD5* with smoking initiation and progression to nicotine dependence. Am. J. Med. Genet..

[21] Plavén-Sigray P., Matheson G.J., Gustavsson P., Stenkrona P., Halldin C., Farde L., Cervenka S. (2018). Is dopamine D1 receptor availability related to social behavior? A positron emission tomography replication study. PLoS ONE.

[22] Lim S., Ha J., Choi S.-W., Kang S.-G., Shin Y.-C. (2011). Association Study on Pathological Gambling and Polymorphisms of Dopamine D1, D2, D3, and D4 Receptor Genes in a Korean Population. J. Gambl. Stud..

[23] Abi-Dargham A., Rodenhiser J., Printz D., Zea-Ponce Y., Gil R., Kegeles L.S., Weiss R., Cooper T.B., Mann J.J., Van Heertum R.L. (2000). Increased baseline occupancy of D2 receptors by dopamine in schizophrenia. Proc. Natl. Acad. Sci. USA.

[24] Comings D.E. (1995). Dopamine D2 Receptor and Tourette’s Syndrome. Arch. Neurol..

[25] Centonze D., Picconi B., Baunez C., Borrelli E., Pisani A., Bernardi G., Calabresi P. (2002). Cocaine and Amphetamine Depress Striatal GABAergic Synaptic Transmission through D2 Dopamine Receptors. Neuropsychopharmacology.

[26] Jiménez K.M., Pereira-Morales A.J., Forero D.A. (2018). A Functional Polymorphism in the *DRD1* Gene, That Modulates Its Regulation by miR-504, Is Associated with Depressive Symptoms. Psychiatry Investig..

[27] Huang W., Ma J.Z., Payne T.J., Beuten J., Dupont R.T., Li M.D. (2007). Significant association of *DRD1* with nicotine dependence. Qual. Life Res..

[28] Yasseen B., Kennedy J.L., Zawertailo L.A., Busto U.E. (2010). Comorbidity between bipolar disorder and alcohol use disorder: Association of dopamine and serotonin gene polymorphisms. Psychiatry Res..

[29] Ishii T., Kimura Y., Ichise M., Takahata K., Kitamura S., Moriguchi S., Kubota M., Zhang M.-R., Yamada M., Higuchi M. (2017). Anatomical relationships between serotonin 5-HT2A and dopamine D2 receptors in living human brain. PLoS ONE.

[30] Zhang L., Hu L., Li X., Zhang J., Chen B. (2014). The *DRD2* rs1800497 polymorphism increase the risk of mood disorder: Evidence from an update meta-analysis. J. Affect. Disord..

[31] Verde Z., Santiago C., González-Moro J.M.R., de Lucas Ramos P., Martín S.L., Bandrés F., Lucia A., Gómez-Gallego F. (2011). ‘Smoking genes’: A genetic association study. PLoS ONE.

[32] Okita K., Mandelkern M.A., London E.D. (2016). Cigarette use and striatal dopamine D2/3 receptors: Possible role in the link between smoking and nicotine dependence. Int. J. Neuropsychopharmacol..

[33] Bahk J.Y., Li S., Park M.S., Kim M.O. (2002). Dopamine D1 and D2 receptor mRNA up-regulation in the caudate–putamen and nucleus accumbens of rat brains by smoking. Prog. Neuro Psychopharmacol. Biol. Psychiatry.

[34] Verplaetse T.L., Morris E.D., McKee S.A., Cosgrove K.P. (2018). Sex differences in the nicotinic acetylcholine and dopamine receptor systems underlying tobacco smoking addiction. Curr. Opin. Behav. Sci..

[35] Novak G., Leblanc M., Zai C., Shaikh S., Renou J., DeLuca V., Bulgin N., Kennedy J.L., Le Foll B. (2010). Association of polymorphisms in the *BDNF*, *DRD1* and *DRD3* genes with tobacco smoking in schizophrenia. Ann. Hum. Genet..

[36] World Health Organization (2019). WHO Global Report on Trends in Prevalence of Tobacco Use 2000–2025.

[37] Rashid R.A., Kanagasundram S., Danaee M., Majid H., Sulaiman A.H., Zahari M.M.A., Guan N.C., Francis B., Husin W.A.I.W., Su T. (2019). The Prevalence of Smoking, Determinants and Chance of Psychological Problems among Smokers in an Urban Community Housing Project in Malaysia. Int. J. Environ. Res. Public Health.

[38] Ryan H., Trosclair A., Gfroerer J. (2012). Adult Current Smoking: Differences in Definitions and Prevalence Estimates—NHIS and NSDUH, 2008. J. Environ. Public Health.

[39] HA A.Y., Ng C., Rusdi A. (2011). Validation of the Malay version of Fagerstrom test for nicotine dependence (FTND-M) among a group of male staffs in a University Hospital. Malays. J. Psychiatry.

[40] Styn M.A., Nukui T., Romkes M., Perkins K., Land S.R., Weissfeld J.L. (2009). The impact of genetic variation in *DRD2* and *SLC6A3* on smoking cessation in a cohort of participants 1 year after enrollment in a lung cancer screening study. Am. J. Med. Genet. Part B Neuropsychiatr. Genet..

[41] Jorm A.F., Henderson A.S., Jacomb P.A., Christensen H., Korten A.E., Rodgers B., Tan X., Easteal S. (2000). Association of smoking and personality with a polymorphism of the dopamine transporter gene: Results from a community survey. Am. J. Med. Genet..

[42] Vandenbergh D.J., Bennett C.J., Grant M.D., Strasser A.A., O’Connor R., Stauffer R.L., Vogler G.P., Kozlowski L.T. (2002). Smoking status and the human dopamine transporter variable number of tandem repeats (VNTR) polymorphism: Failure to replicate and finding that never-smokers may be different. Nicotine Tob. Res..

[43] Timberlake D.S., Haberstick B.C., Lessem J.M., Smolen A., Ehringer M., Hewitt J.K., Hopfer C. (2006). An association between the *DAT1* polymorphism and smoking behavior in young adults from the national longitudinal study of adolescent health. Health Psychol..

[44] Ling D., Niu T., Feng Y., Xing H., Xu X. (2004). Association between polymorphism of the dopamine transporter gene and early smoking onset: An interaction risk on nicotine dependence. J. Hum. Genet..

[45] Breitling L.P., Muller H., Illig T., Rujescu D., Winterer G., Dahmen N., Nitz B., Raum E., Rothenbacher D., Brenner H. (2011). Dopamine-related genes and spontaneous smoking cessation in ever-heavy smokers. Pharmacogenomics.

[46] Diakonova A.T., Pavlova N.I., Solovyeva N.A., Varlamova M.A., Alexandrova T.N., Kurtanov K.A. (2018). Molecular-genetic analysis of the connection of the *SLC6A3* gene with nicotine addiction in Yakutia. Yakut Med. J..

[47] Li S., Wang Q., Pan L., Li H., Yang X., Jiang F., Zhang N., Han M., Jia C. (2016). The association of dopamine pathway gene score, nicotine dependence and smoking cessation in a rural male population of Shandong, China. Am. J. Addict..

[48] Robles A.I., Yang P., Jen J., McClary A.C., Calhoun K., Bowman E.D., Vähäkangas K., Greathouse K.L., Wang Y., Olivo-Marston S. (2014). A *DRD1* polymorphism predisposes to lung cancer among those exposed to secondhand smoke during childhood. Cancer Prev. Res..

[49] Hirasawa-Fujita M., Bly M.J., Ellingrod V.L., Dalack G.W., Domino E.F. (2017). Genetic Variation of the Mu Opioid Receptor (OPRM1) and Dopamine D2 Receptor (*DRD2*) is Related to Smoking Differences in Patients with Schizophrenia but not Bipolar Disorder. Clin. Schizophr. Relat. Psychoses.

[50] Comings D.E., Ferry L., Bradshaw-Robinson S., Burchette R., Chiu C., Muhleman D. (1996). The dopamine D2 receptor (*DRD2*) gene: A genetic risk factor in smoking. Pharmacogenetics.

[51] Noble E., Jeor S.S., Ritchie T., Syndulko K., Jeor S.S., Fitch R., Brunner R., Sparkes R. (1994). D2 dopamine receptor gene and cigarette smoking: A reward gene?. Med. Hypotheses.

[52] Wilcox C.S., Noble E.P., Oskooilar N. (2011). *ANKK1*/*DRD2* locus variants are associated with rimonabant efficacy in aiding smoking cessation: Pilot data. J. Investig. Med..

[53] Hamajima N., Ito H., Matsuo K., Saito T., Tajima K., Ando M., Yoshida K., Takahashi T. (2002). Association between Smoking Habits and Dopamine Receptor D2 Taql A A2 Allele in Japanese Males: A Confirmatory Study. J. Epidemiol..

[54] Mayer O., Seidlerová J., Černá V., Kučerová A., Bruthans J., Vágovičová P., Vaněk J., Timoracká K., Wohlfahrt P., Filipovský J. (2015). The *DRD2*/*ANKK1 Taq1A* polymorphism is associated with smoking cessation failure in patients with coronary heart disease. Pers. Med..

[55] Huang C.-L., Ou W.-C., Chen P.-L., Liu C.-N., Chen M.-C., Lu C.-C., Chen Y.-C., Lin M.-H., Huang C.-S. (2015). Effects of Interaction Between Dopamine D2 Receptor and Monoamine Oxidase A Genes on Smoking Status in Young Men. Biol. Res. Nurs..

[56] Munafò M.R., Johnstone E.C., Murphy M.F.G., Aveyard P. (2009). Lack of association of *DRD2* rs1800497 (*Taq1A*) polymorphism with smoking cessation in a nicotine replacement therapy randomized trial. Nicotine Tob. Res..

[57] Breitling L.P., Twardella R., Hoffmann M.M., Witt S.H., Treutlein J., Brenner H. (2010). Prospective association of dopamine-related polymorphisms with smoking cessation in general care. Pharmacogenomics.

[58] Batra A., Gelfort G., Bartels M., Smoltczyk H., Buchkremer G., Riess O., Schöls L. (2000). The dopamine D2 receptor (*DRD2*) gene—A genetic risk factor in heavy smoking?. Addict. Biol..

[59] Ton T.G., Rossing M.A., Burke L.M., Srinouanprachan S., Wicklund K., Farin F. (2007). Genetic polymorphisms in dopamine-related genes and smoking cessation in women: A prospective cohort study. Behav. Brain Funct..

[60] Morton L.M., Wang S.S., Bergen A.W., Chatterjee N., Kvale P., Welch R., Yeager M., Hayes R.B., Chanock S.J., Caporaso N.E. (2006). *DRD2* genetic variation in relation to smoking and obesity in the Prostate, Lung, Colorectal, and Ovarian Cancer Screening Trial. Pharm. Genom..

[61] Radwan G., Loffredo C.A., El Setouhy M.A., Hamid M.A., Israel E.J., Mohamed M.K. (2010). Waterpipe Smoking and The *DRD2*/*ANKK1* Genotype. J. Egypt. Public Health Assoc..

[62] Radwan G.N., Setouhy M.E., Mohamed M.K., Hamid M.A., Israel E., Azem S.A., Kamel O., Loffredo C.A. (2007). *DRD2*/*ANKK1* TaqI polymorphism and smoking behavior of Egyptian male cigarette smokers. Nicotine Tob. Res..

[63] Suriyaprom K., Tungtrongchitr R., Harnroongroj T. (2013). Impact of COMT Val 108/158 Met and *DRD2* Taq1B gene polymorphisms on vulnerability to cigarette smoking of Thai males. J. Mol. Neurosci..

[64] Tomlinson G.E., Tsoh J.Y., De Moor C.A., Cinciripini L.G., Minna J.D., Cinciripini P.M., Wetter D.W. (2004). The effects of the *DRD2* polymorphism on smoking cessation and negative affect: Evidence for a pharmacogenetic effect on mood. Nicotine Tob. Res..

[65] Ragia G., Iordanidou M., Giannakopoulou E., Tavridou A., Manolopoulos V.G. (2013). Association of *DRD2* TaqIA and DβH -1021C>T Gene Polymorphisms with Smoking Initiation and their Interaction with Serotonergic System Gene Polymorphisms. Curr. Pharm. Pers. Med..

[66] Hill K.G., Hawkins J.D., Catalano R.F., Abbott R.D., Guo J. (2005). Family influences on the risk of daily smoking initiation. J. Adolesc. Health.

[67] McGee C.E., Trigwell J., Fairclough S.J., Murphy R.C., Porcellato L., Ussher M., Foweather L. (2015). Influence of family and friend smoking on intentions to smoke and smoking-related attitudes and refusal self-efficacy among 9–10 year old children from deprived neighbourhoods: A cross-sectional study. BMC Public Health.

[68] Saari A.J., Kentala J., Mattila K.J. (2014). The smoking habit of a close friend or family member—How deep is the impact? A cross-sectional study. BMJ Open.

[69] Pizent A., Lazarus M., Kovačić J., Tariba Lovaković B., Brčić Karačonji I., Živković Semren T., Sekovanić A., Orct T., Branović-Čakanić K., Brajenović N. (2020). Cigarette Smoking during Pregnancy: Effects on Antioxidant Enzymes, Metallothionein and Trace Elements in Mother-Newborn Pairs. Biomolecules.

