# Supplementary material for: Smoking Genes: A Case–Control Study of Dopamine Transporter Gene (SLC6A3) and Dopamine Receptor Genes (DRD1, DRD2 and DRD3) Polymorphisms and Smoking Behaviour in a Malay Male Cohort
Source: Biomolecules. 2020 Dec 3;10(12):1633. doi: 10.3390/biom10121633 (PMC7761729; doi:10.3390/biom10121633)
Supplement: Supplementary file 1 [file biomolecules-10-01633-s001.pdf]

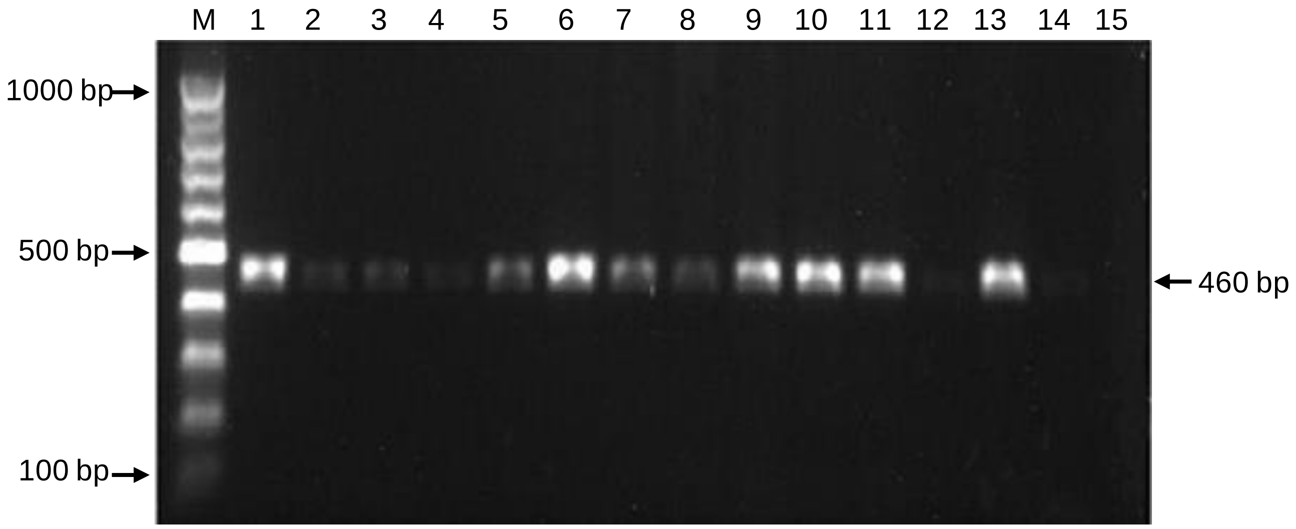

Figure S1: PCR product (460 bp) of *SLC6A3* gene (rs27072).

Lane M: 100 bp DNA ladder; Lane 1-14: 460 bp PCR product; Lane 15: Negative control.

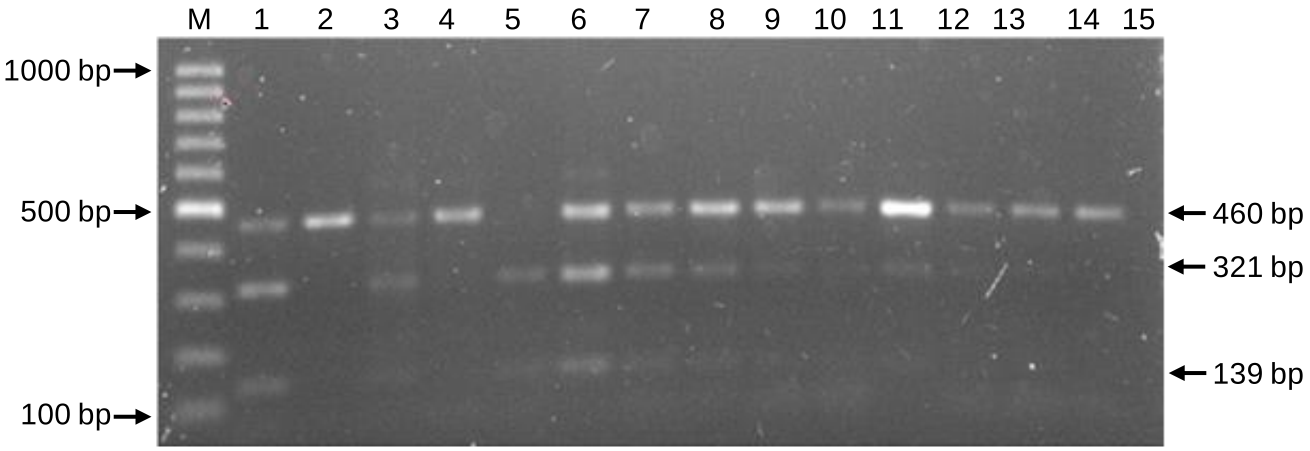

Figure S2: RFLP result of *SLC6A3* gene (rs27072).

Lane M: 100 bp DNA ladder; Lane 1, 3, 6-13: Heterozygous mutant (139 bp, 321 bp and 460 bp); Lane 2, 4, 14: Homozygous mutant (460 bp); Lane 5: Homozygous wild type (139 bp and 321 bp); Lane 15: Negative control.

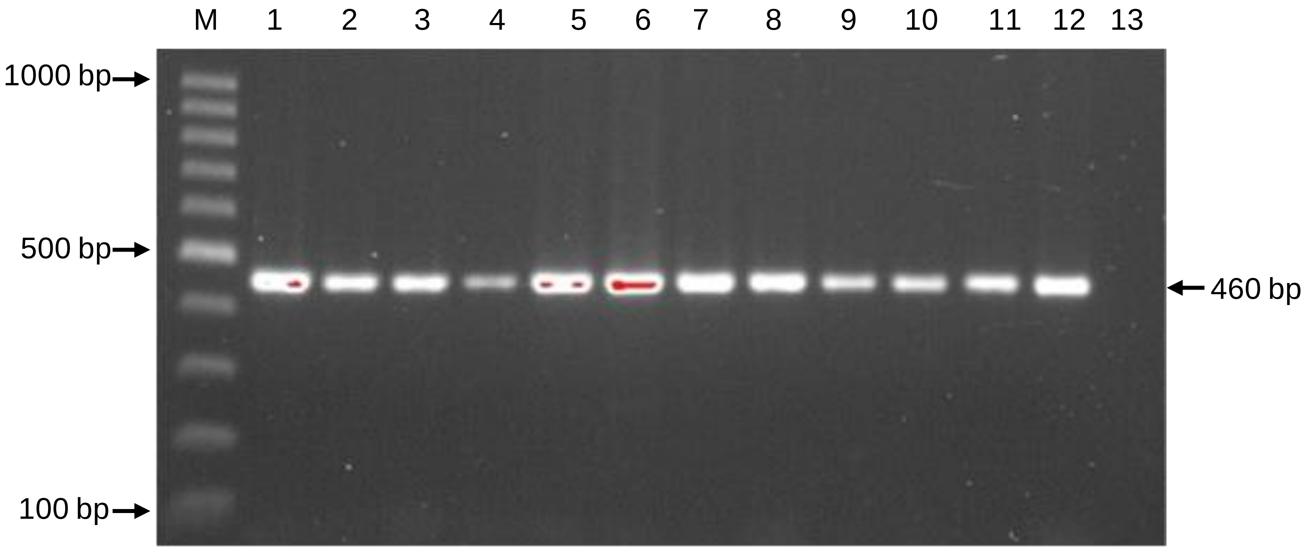

Figure S3: PCR product (460 bp) of *DRD1* gene (rs686).

Lane M: 100 bp DNA ladder; Lane 1-12: 460 bp PCR product; 13: Negative control.

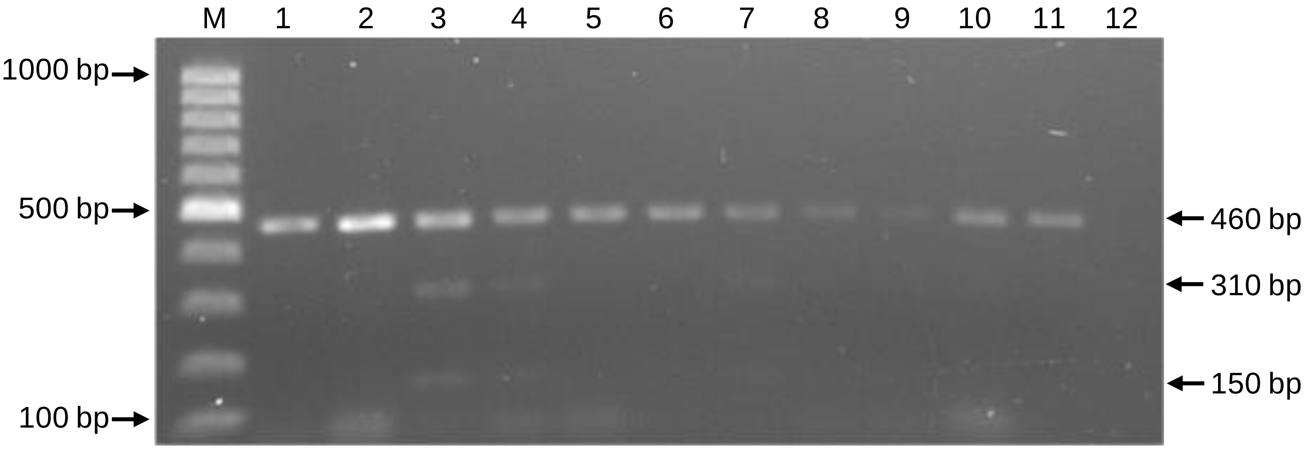

Figure S4: RFLP result of *DRD1* gene (rs686).

Lane M: 100 bp DNA ladder; Lane 1, 2, 5, 6, 8-11: Homozygous mutant (460 bp); Lane 3, 4, 7: Heterozygous mutant (150 bp, 310 bp and 460 bp); Lane 12: Negative control.

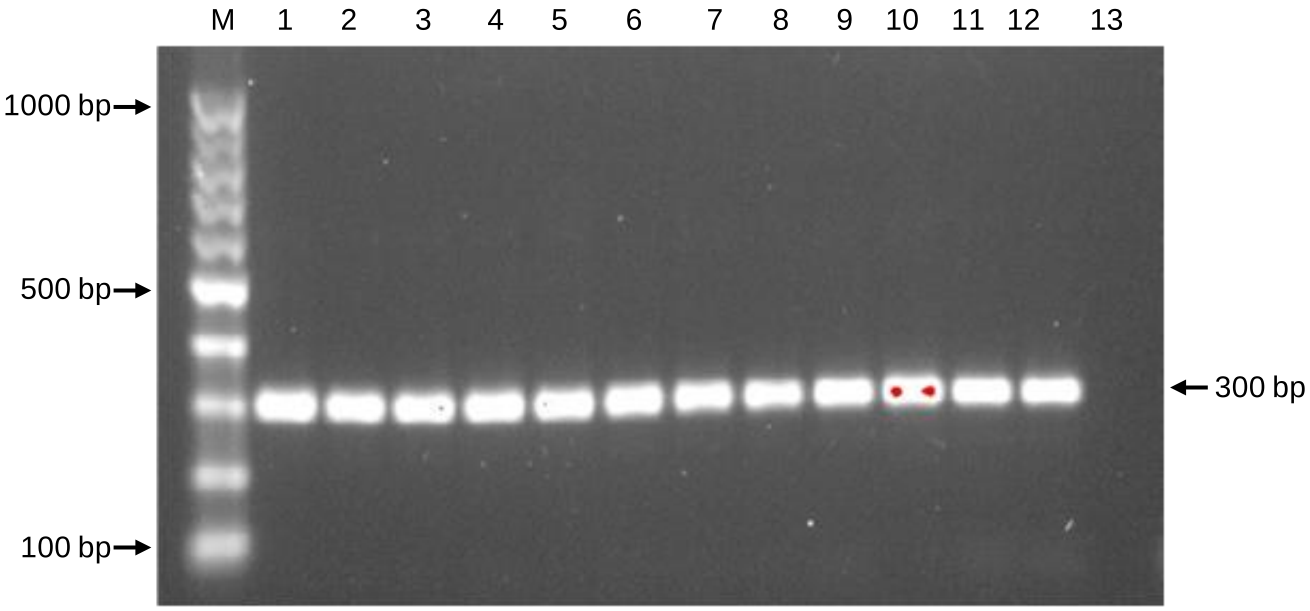

Figure S5: PCR product (300 bp) of *DRD2* gene (rs1800497).

Lane M: 100 bp DNA ladder; Lane 1-12: 300 bp PCR product; Lane 13: Negative control.

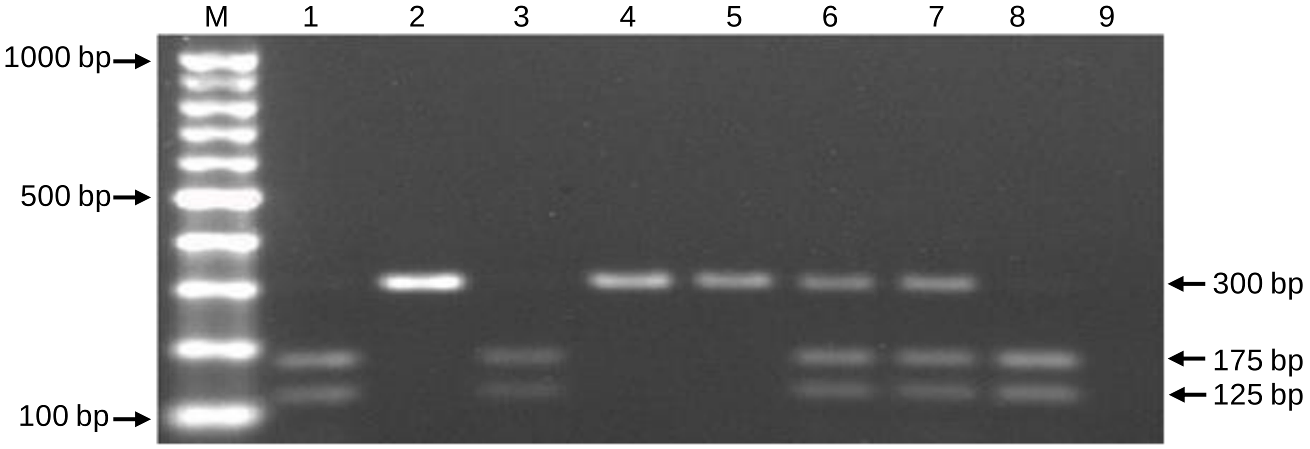

Figure S6: RFLP result of *DRD2* gene (rs1800497).

Lane M: 100 bp DNA ladder; Lane 1, 3, 8: Homozygous wild type (125 bp and 175 bp); Lane 2, 4, 5: Homozygous mutant (300 bp); Lane 6, 7: Heterozygous mutant (125 bp, 175 bp and 300 bp); Lane 9: Negative control.

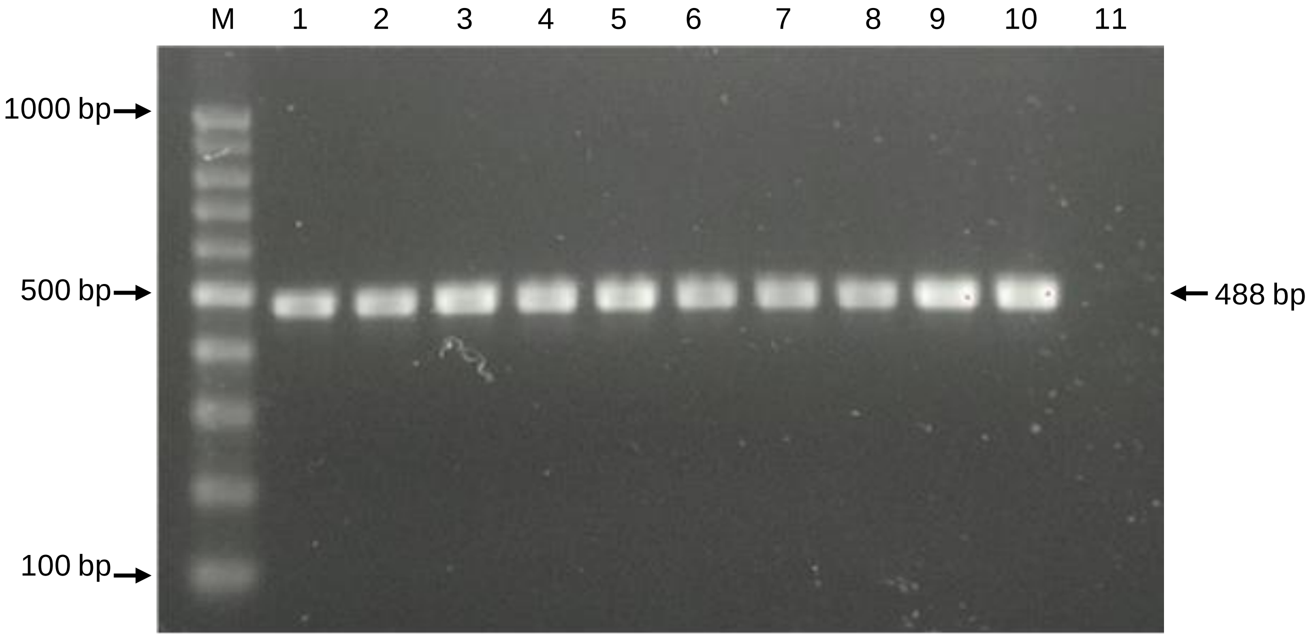

Figure S7: PCR product (488 bp) of *DRD3* (rs7653787).

Lane M: 100 bp DNA ladder; 488 bp PCR product; Lane 11: Negative control.

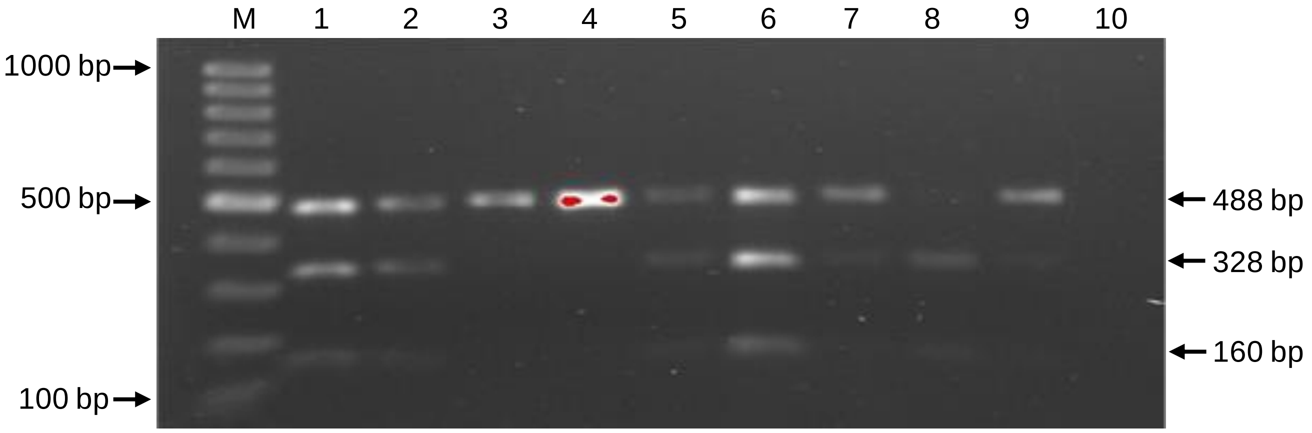

Figure S8: RFLP result of *DRD3* gene (rs7653787).

Lane M: 100 bp DNA ladder; Lane 1, 2, 5-7, 9: Heterozygous mutant (160 bp, 328 bp and 488 bp); Lane 3, 4: Homozygous mutant (488 bp); Lane 8: Homozygous wild type (160 bp and 328 bp); Lane 10: Negative control.

A)

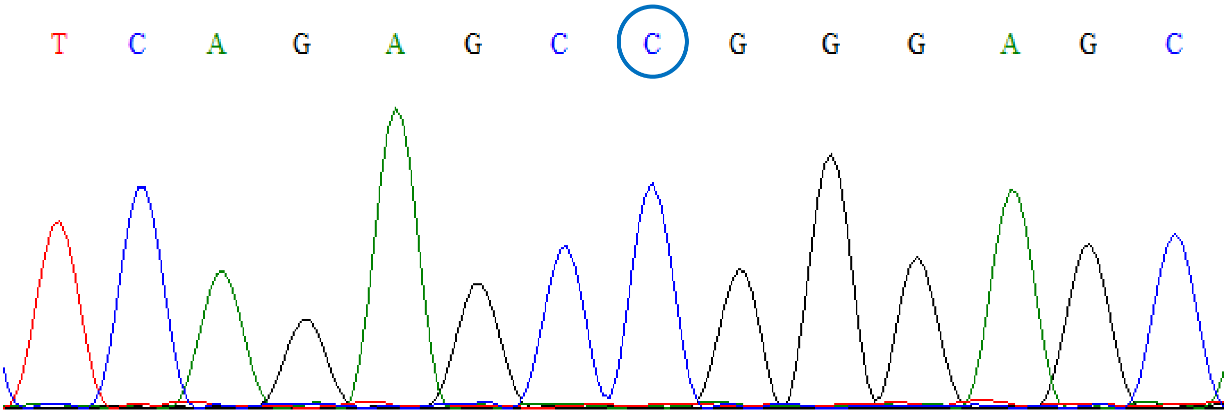

B)

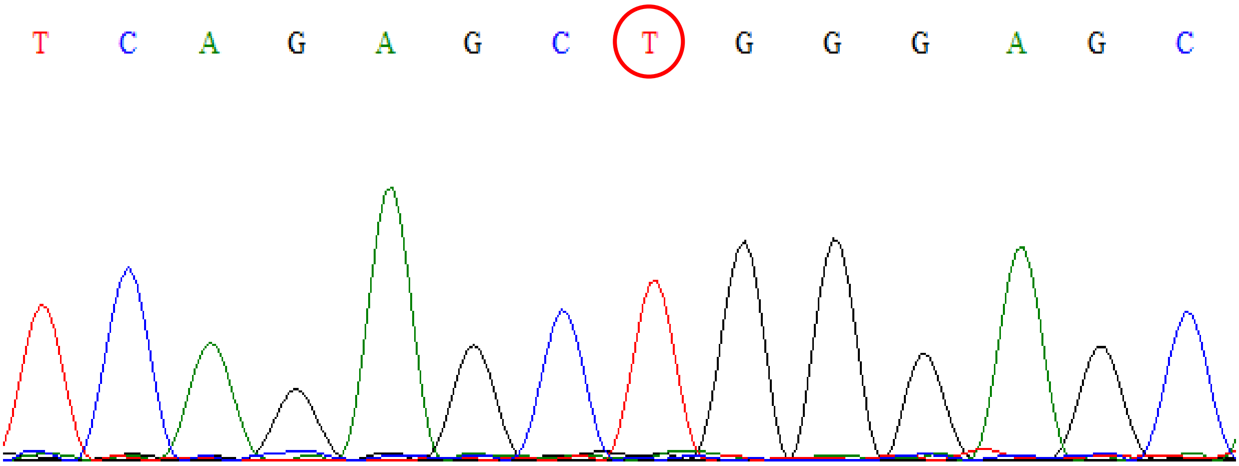

Figure S9: Sequencing analysis of *SLC6A3* gene (rs27072) (A) the chromatograms of *SLC6A3* gene (rs27072) homozygous wild type sequence and (B) *SLC6A3* gene (rs27072) heterozygous mutant sequence.

A)

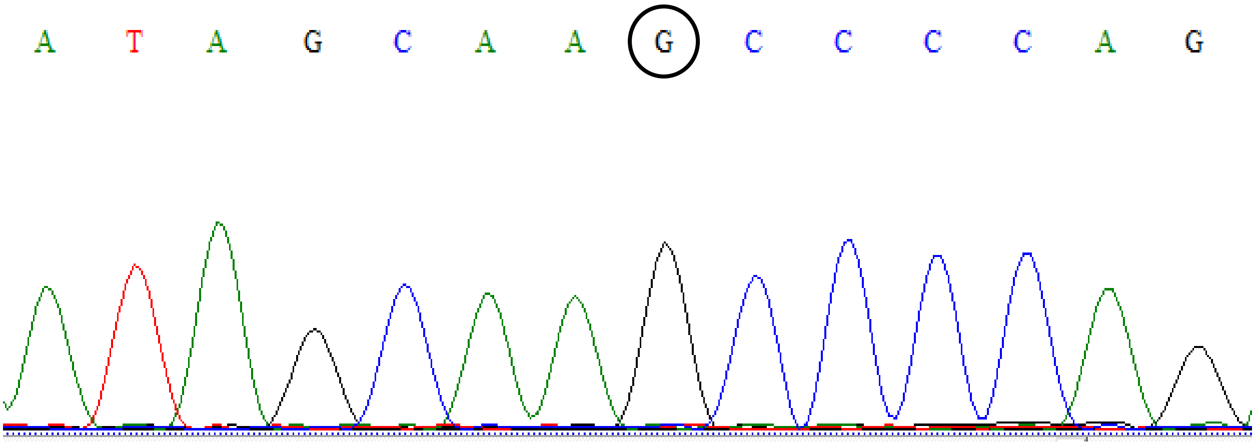

B)

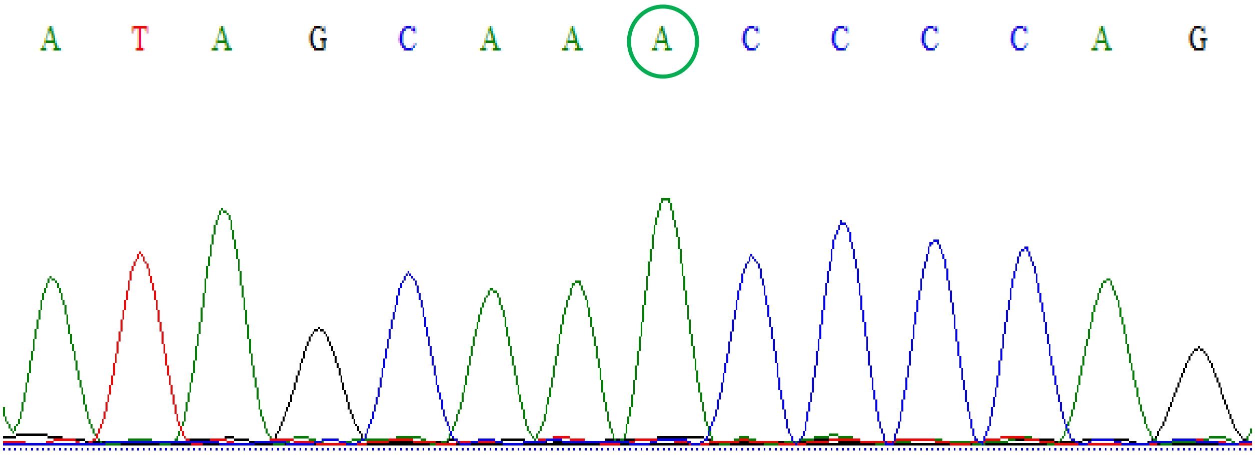

Figure S10: Sequencing analysis of *DRD1* (rs686) (A) the chromatograms of *DRD1* gene (rs686) homozygous wild type sequence and (B) *DRD1* gene (rs686) heterozygous mutant sequence.

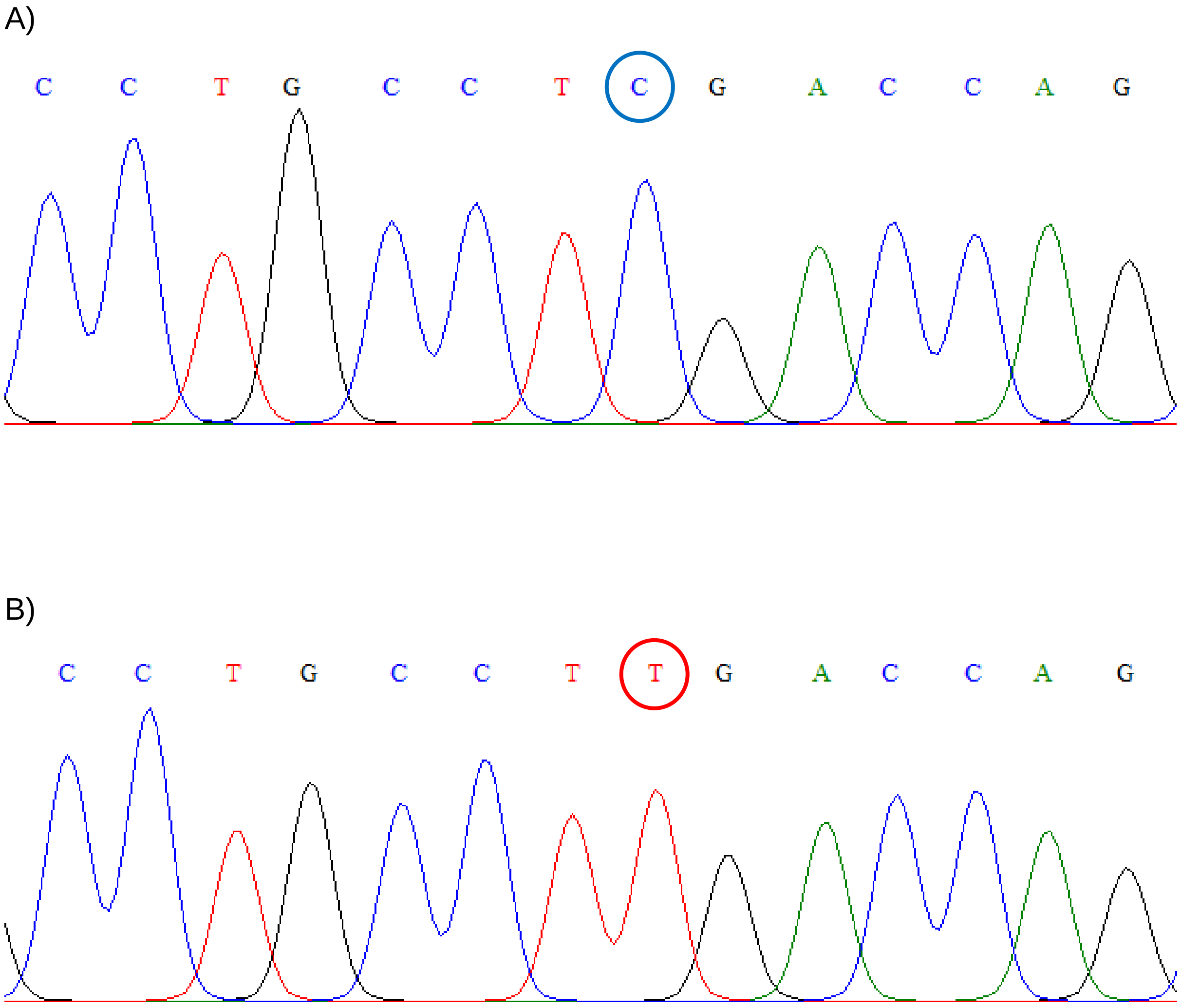

Figure S11: Sequencing analysis of *DRD2* (rs1800497) (A) the chromatograms of *DRD2* gene (rs1800497) homozygous wild type sequence and (B) *DRD2* gene (rs1800497) heterozygous mutant sequence.

A)

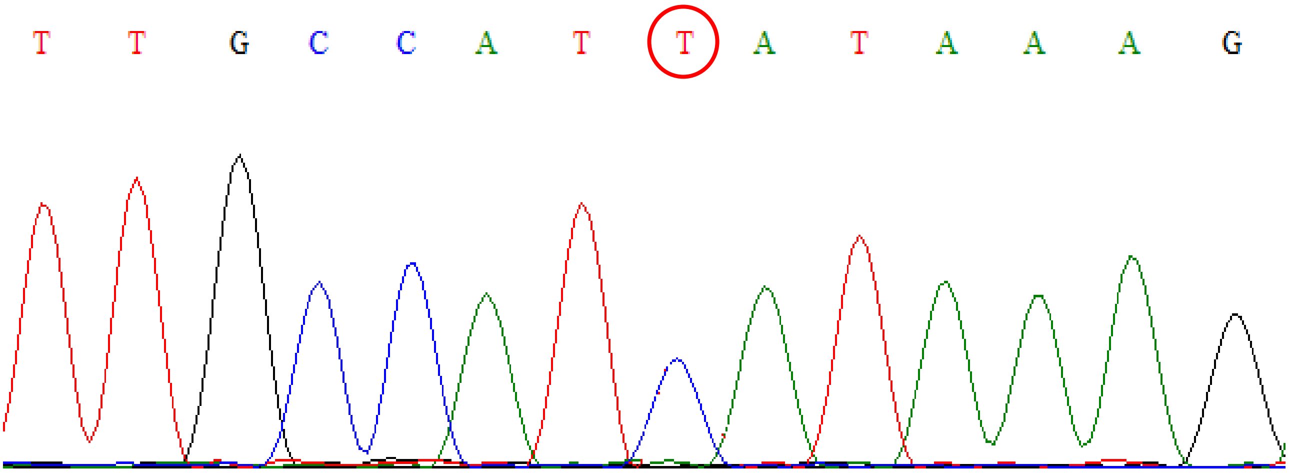

B)

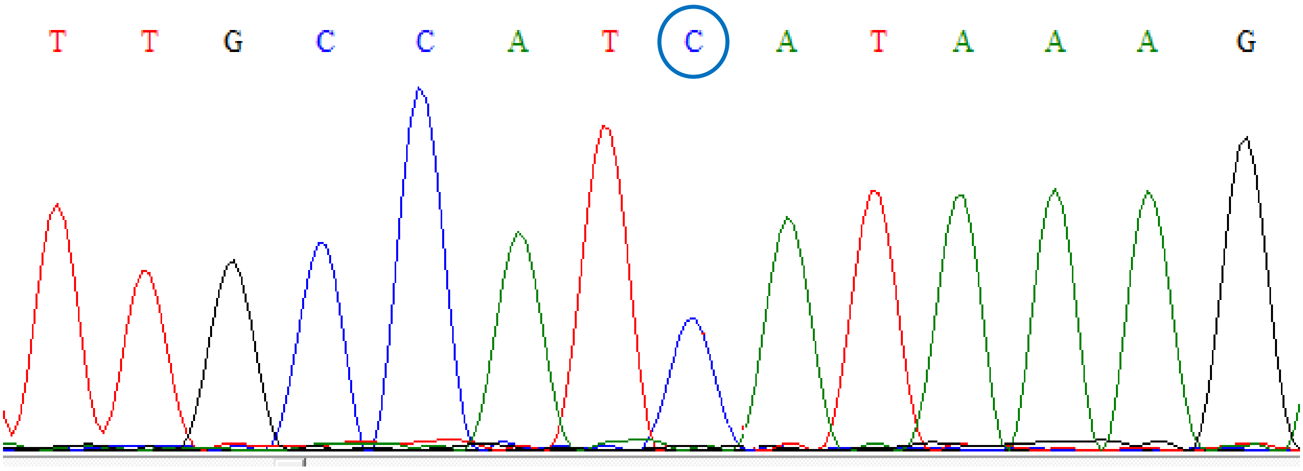

Figure S12: Sequencing analysis of *DRD3* (rs7653787) (A) The chromatograms of *DRD3* gene (rs7653787) homozygous wild type sequence and (B) *DRD3* gene (rs7653787) heterozygous mutant sequence.
